# On the Use of Opium in Fever

**Published:** 1802-07

**Authors:** 

**Affiliations:** Abingdon


### *On the Use of Opium in Fever. Communicated by* Mr. Atkinson, *of Abingdon.*

THE influence of contagious diſeaſes, and their peculiar operation on mankind, have excited the attention and rouſed the ſagacity of the Faculty from time immemorial. The noxious powers which produce fever are aſcertained to be human effluvia, generated commonly in the confined miſerable huts of penury and wretchedneſs, from the putrid atmoſphere of marſhes, and from the damp unventilated cells of impriſonment. Animals expoſed to this malignant vapor, feel it abſorbed into the ſyſtem, and are affected with its well-known attendant conſequences. He who is ſo ſituated as to witneſs the ravages which a peſtilential diſorder commits amongſt the poor, and who knows the difficulty of ſtemming its ſweeping violence amidſt poverty and filth, will coincide with me, that in towns where extenſive manufactories are kept, certain regulations and improvements, rigidly impoſed, would be the means of ſaving from the jaws of Death a conſiderable portion of our fellow-creatures. The numbers which die annually from neglect, and from the cruel parſimony of managers of pariſhes, are almoſt incredible. I have had many opportunities of exploring theſe dreary abodes of diſtreſs, and have found the inefficacy of medicine when individuals have been reduced by ſtarvation and diſeaſe. Theſe conſiderations induced ſome humane profeſſors of the healing art, to write upon this important ſubject, and to ſtate the moſt probable mode of preventing or weakening the force force of epidemic fevers: But it is to be lamented, fuch benevolent, well-meant exertions are too often difregarded by the reputed guardians of a people's welfare.

Refpecting typhus fevers, various remedies have been propofed; but fince the writings of the illuftrious Brown illumined the medical world, the treatment has been better underftood; the art had been long buried under the accumulated, enveloping doctrines of different ages, till his Elementa Medicinæ appeared, and ftripped her of all her antique, fanciful trappings, which were only calculated to puzzle and confound the ftudent. Can there refult any good from intricate theory and contradictory practice? The more SIMPLE the theory, does it not appear the moft *rational?* Muft not the fimpleft practice,* when fupported by juft principles and experience, be the moft extenfively ufeful, and the leaft dangerous in its application? Till lately, has the great efficacy of opium been rightly known; or has its properties † been juftly inveftigated? Dr. Brown, and his difciples, Drs. Blane and Campbell, have given opium in low fevers with general fuccefs, for it fometimes fuperfedes the ufe of the Peruvian bark. I was fanguine to try the virtues of this valuable medicine, *without any auxiliary*, in the above difeafe; and the following is a Sketch of a Cafe, amongft

* Since the perufal of Mr. Cook's paper on Acetous Ablution, (No. 36) I feel much fatisfaction in ftating, that two cafes of Synochus, which lately occurred to me, have been decifively remedied by the above excellent practice. One patient was a poor girl, twelve years old. She had been three days ill before I faw her. Her pulfe was flow and fmall; perfpiration profufe, with a fhooting pain in her head, fometimes occafioning flight delirium. Her tongue was dry and furred. She was rubbed all over with common vinegar, and particularly well over the head and neck, three or four times a day. She took no medicines internally, as I wifhed fairly to afcertain the effects of the external application. I found fhe recovered fooner than thofe who had taken the common medicines, which too arbitrary cuftom prefcribes. The other patient was younger, on whom the fame mode of treatment was fuccefsfully adopted, excepting a mixture of aq. puræ, and a few drops of tinct. lavend. comp. were ordered, to fatisfy the importunity of friends, who imagined the vinegar externally applied too infignificant to effect a cure. The difficulty which is immediately ftarted on recommending to a patient the means which Dr. Currie has found fo beneficial in fever, makes it impoffible for me to fpeak of its utility from experience. People feel fuch a *horror* at being taken out of a warm bed to be dafhed over with cold water, it is probable fuch practice will never be univerfally employed.

† A few days ago, for the fake of experiment, I fwallowed four grains of opium; and the confequence was, I had more vivacity and better fpirits than ufual, but had not the leaft difpofition to fleep. I never took opium before. I embrace this opportunity of thanking Mr. Ward for the pleafure received from the perufal of his excellent Differtation on the Modus Operandi of Opium, inferted in the Medical Journal.

 others, others, which terminated favorably, without leaving any œdematous fwelling of the extremities behind.

R. Lawrence, of Wooton, Bucks, a boy aged fifteen, was taken ill of typhus fever on the 12th of May laft. It came on with chillinefs and laffitude. The face was flufhed; the eyes red and watery; tongue furred; and he complained of univerfal tremors. An emetic was adminiftered in the evening, which operated well. The day following, an ulceration of the throat appeared. Accordingly, blifters were applied with a gargle of infus. rofæ et tinct. myrrhæ, directed to be ufed frequently. Ten drops of tinct. opii were given every four hours; but on the 16th, every fymptom was aggravated. The face was fuffufed with purple; he had great anxiety and moaning, and a low ftupid delirium fupervened. 17th, The pulfe grew ftrong and full, and the inflammation of the uvula and tonfils abated. The drops were now increafed to fifteen every four hours. The two fucceeding days he recovered faft, and equal parts of æther. et tinct. opii were prefcribed, to be taken as before. With this fimple treatment, in ten days from the commencement of the fever, his ufual good health and ftrength returned. It may be neceffary to add, that an aperient medicine was now and then interpofed, to obviate the coftivenefs occafioned by the ufe of opium.

The fubjoined cafe is an additional evidence of the efficacy of Opium in contagious fevers of a putrid tendency. But there exifts a blameable fault with many medical men, who refufe to defcribe fatal cafes; they frequently regard fuch, as proofs of their incapacity or unfkilful conduct. Continually advertizing fuccefsful ones, is certainly tantamount to faying, "We are infallible." But nothing can be more inftructive, or of more fterling utility, than a relation of the impediments daily occurring in medical practice. We are warned by the fatal termination of difeafes; but a continued fucceffion of fortunate events leads to indolence, and we dream on till all difficulties vanifh, like fpectres before the magician's wand. At prefent, I believe, that fimple uncombined fevers will uniformly yield to opium, judicioufly given; but when one difeafe is complicated with others equally alarming, the prognofis muft always be doubtful. Two foldiers of the 30th regiment of foot, juft arrived from the campaign in Egypt, and on their march to the north of England, were left behind, labouring under fever. J. Spencer, one of them, was taken ill four days before he came under my care, which was on the 16th of May, 1802. By being conveyed here on the baggage cart, the difeafe was confiderably increafed. He had, a month before, been attacked with with ſymptoms of peripneumony. His breathing was now difficult; expectoration bad, and ſtreaked with blood; had acute pain in the whole cheſt; his face was livid, tongue dry, pulſe low, and the characteriſtics of typhus were ſtrongly marked. He took a pill of one grain of opium and four of camphor every four hours, and a mixture of lac. ammoniaci, acet. ſcillæ, ſyr. et tinct. digital. in the intervals. A bliſter was alſo applied on the ſternum. On the 18th, he ſeemed relieved; his pulſe full and ſtrong, owing, I conceive, to the ſtimulating effects of the opium and camphor. 19th, The bliſter roſe well. In the evening he had watching and delirium. An anodyne draught of tinct. opii gt. lx. joined with cathartic infuſions, was given. 20th, Had had a few hours ſleep. As the bowels were bound, an enema was exhibited, which produced one copious evacuation. At night the anodyne draught, with a few drops of æther, was repeated. 21ſt, Towards morning his breathing became more laborious, he moaned conſtantly, and death ſoon cloſed the cataſtrophe.

T. Macgowan was ſeized about the ſame time as his comrade. In the firſt inſtance they both had taken emetics. This man had typhus fever in its moſt deſperate ſhape. He had great hurry and confuſion of intellect, with low muttering delirium; his pulſe was ſmall and unequal. He alſo took the camphor and opium every four hours. For two days the diſeaſe appeared ſtationary, and then gradually gave way. The ſame medicine was continued till his health was ſo far eſtabliſhed, that he could purſue his route to the regiment with ſafety.

There is no novelty in applying opium in low nervous fevers; and in the preceding favourable inſtances, can the recovery be aſcribed to any other remedy? When combined with cordials and the bark, the efficient article cannot poſſibly be known; it may be attributed to one, or to the joint operation of them all. Is it not now ſufficiently known, that opium will cure thoſe low fevers ſo predominant amongſt the poor? Or is the bark the only drug to be depended on? Does not the bark very frequently operate by the bowels? And is not the appearance of a diarrhœa of the utmoſt conſequence, nay of the greateſt danger, to the patient? Is not the aid of opium then abſolutely neceſſary? it will check the progreſs of diarrhœa, of putreſcency, and of gangrene. But I ſhall reſume this ſubject in ſome future communication.

*June* 10, 1802.

