# Erratum

**Published:** 1802-08-01

**Authors:** 


### ERRATUM.

Page 52, line 3, for *Bucks*, read *Berks*.

